# Levels of intervention and support for newly presenting clients with eating disorders

**DOI:** 10.1192/j.eurpsy.2022.1483

**Published:** 2022-09-01

**Authors:** A. Grau, C. Evans, J.C. Medina, C. Paz

**Affiliations:** 1 ITA Mental Health, Avenir Unit, Barcelona, Spain; 2 PSYCTC.org, Research, Aime la Plagne, France; 3 University of Sheffield, Psychology, Sheffield, United Kingdom; 4 Universidad de Las Américas, Ecuador, Psychology, Aime, France; 5 Universitat Oberta de Catalunya, Psychology, Barcelona, Spain; 6 Universidad de Las Américas, Ecuador, Psychology, Quito, Ecuador

**Keywords:** Eating Disorders, levels of care, personalised care, Psychotherapy

## Abstract

**Introduction:**

Clients with Eating Disorders may be treated as inpatients (IP), day hospital (DH) or community patients (Ambu). The ITAMITED study is following to treatment termination (or end of October 2025) all new clients with EDs presenting between November 2017 and October 2020 to eight centres in Spain.

**Objectives:**

To describe to what extent initial care levels (IP, DH, Ambu) are associated with gender, age, social relationships, ED diagnosis Body Mass Index (BMI) and baseline medication.

**Methods:**

The study is exploratory/descriptive, practice-based evidence (PBE). Consecutive new clients were approached for participation. Inclusion criteria were a diagnosis of an ED and opting in to treatment.

**Results:**

The only variables *not* showing a statistically significant relationship with level were gender (*no* relationship), diagnosis of bulimia and use of prescribed psychostimulant medication (which was rare). OP care was associated with older age which did not discriminate between DH and IP. Similarly, being in a relationship was associated with OP care but didn’t differentiate between DH and OP. Relationships with ED diagnosis other than AN type I were complex but significant. Relationships with AN type I, BMI and being on antipsychotics, antidepressants, anxiolytics, mood stabilisers and a catch-all category of all other medication all showed an ordered relationship IP > DH > Ambu. The most powerful relationships were with BMI and diagnosis of AN type I.

**Figure:**
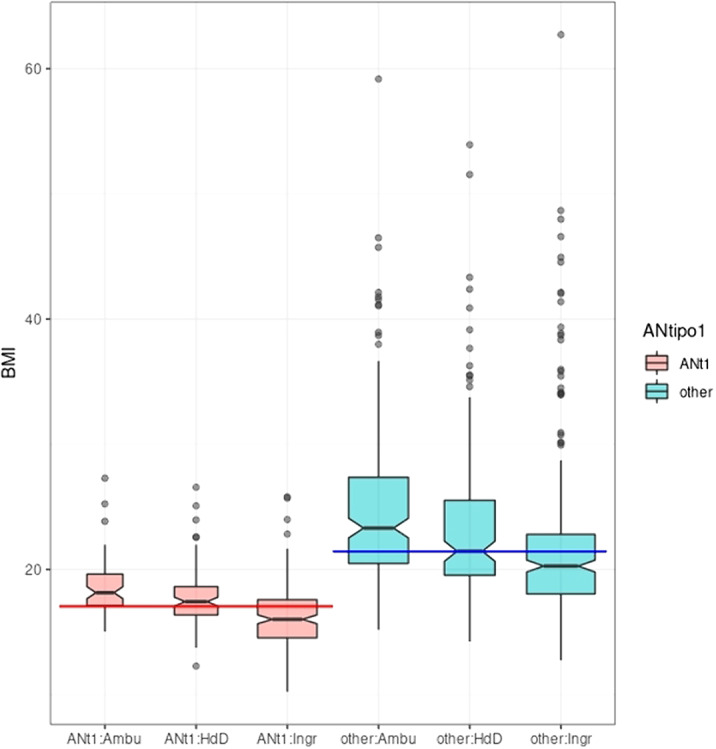

**Conclusions:**

Initial level of care is associated with many factors and strongly with many of them. This will complicate the analyses of trajectories of change but reflects the heterogeneity of this client group.

**Disclosure:**

I am Clinical Director of ITA but analyses are prespecified. Evans, Medina and Paz are not remunerated by ITA nor related to ITA or any of its employees they would counter any pressure on analyses or reporting that might arise from my position.

